# 肺原发恶性纤维组织细胞瘤20例

**DOI:** 10.3779/j.issn.1009-3419.2011.05.06

**Published:** 2011-05-20

**Authors:** 崇标 黄, 亮 辛, 焱 崔, 大亮 齐

**Affiliations:** 300060 天津，天津市肿瘤防治重点实验室，天津医科大学附属肿瘤医院高级病房 Department of VIP Ward, Cancer Institute and Hospital of Tianjin Medical University, Tianjin Key Laboratory of Cancer Treatment and Prevention, Tianjin 300060, China

**Keywords:** 恶性纤维组织细胞瘤, 预后, 多因素分析, Malignant fibrous histiocytoma, Prognosis, Multivariate analysis

## Abstract

**背景与目的:**

肺原发恶性纤维组织细胞瘤是一种罕见的肿瘤。本文研究肺原发恶性纤维组织细胞瘤的临床特点及预后影响因素。

**方法:**

回顾性分析肺原发恶性纤维组织细胞瘤20例的临床特点、治疗方式及生存情况等资料，采用SPSS 16.0统计学软件，用*Kaplan-Meier*法及*COX*回归分析性别、肿瘤大小、肿瘤位置及化疗对术后生存期的影响。

**结果:**

肺原发MFH的临床症状主要有咳嗽咳痰、痰中带血、胸痛、发热和胸闷。本组患者1年生存率为55.0%，2年生存率为25.0%，死亡原因多为局部复发及远处转移。肿瘤的大小和位置是肺原发MFH总生存率的独立预后因素。

**结论:**

肺恶性纤维细胞瘤恶性程度高，预后差。临床治疗以手术为主，术后辅助化疗疗效尚不确切，肿瘤大小及位置和预后相关。

## Abstract (en)

**Background and objective:**

Primary malignant fibrous histiocytoma (MFH) of the lung is a type of rare sarcoma which showed a lack of detailed guidance about treatment and prognosis. The clinical features, treatment methods and prognosis of primary MFH of the lung were analyzed by this study to reveal some information of this disease.

**Methods:**

The clinical data and survival state of 20 patients with primary pulmonary MFH treated in Cancer Institute and Hospital of Tianjin Medical University were collected. SPSS 16.0 software was used for statistical analysis. *Kaplan-Meier* method was applied to figure out whether gender, size, site and post-operative chemotherapy correlated to prognosis. *COX* regression was employed for multivariate prognostic analysis to find the unattached prognostic factors. Statistical significance was considered at *P* < 0.05.

**Results:**

The clinical manifestations of primary pulmonary MFH mainly included cough, blood-stained sputum, chest pain, fever and chest distress. The one-year and two-year overall survival rates were 55.0% and 25.0%, respectively. Most cases died of local recurrence and distant metastasis. Multivariate *COX* regression analysis showed tumor size and location type were unattached prognostic factors. The patients with tumors smaller than 5 cm had a median survival of 27 months, while larger than 5 cm group's median survival was 8 months. The median survival of central tumor and the peripheral tumor were 6 months and 23 months respectively.

**Conclusion:**

Primary pulmonary MFH was a type of highly malignant sarcoma which had poor prognosis. The main effective treatment was surgery, yet the role of postoperative adjuvant chemotherapy in the whole course of treatment was undefined. Small mass size and peripheral type might be correlated to relatively better prognosis.

恶性纤维组织细胞瘤（malignant fibrous histiocytoma, MFH）起源于间叶组织，通常好发与四肢和腹膜后，以席纹状排列的多形性纤维细胞和组织细胞为特点，是成人最常发生的软组织肉瘤之一，约占所有软组织肉瘤的10%^[1]^。虽然75%深部MFH可以转移到肺部^[2]^，但是肺原发的MFH实为罕见，约占肺恶性肿瘤的0.02%-0.30%^[3]^。检索文献发现国内仅报道百余例^[4, 5]^。

## 1 材料与方法

### 1.1 一般情况

天津医科大学附属肿瘤医院胸外科自1986年3月-2008年12月共收治肺原发恶性纤维组织细胞瘤28例，其中病历资料完整的20例，男性15例，女性5例。年龄范围40岁-76岁，中位年龄61.6岁。病例入组要求：有完整的临床资料，包括一般情况、病理诊断及治疗方式，能随访至患者目前生存状态或者死亡时间。

### 1.2 方法

全部患者均经手术病理诊断为肺原发MFH。20例患者中16例行根治性切除术（其中肺叶切除术14例，全肺切除术2例），3例行姑息性切除术，1例行开胸切检术。9例行术后辅助化疗，方案为铂类联合阿霉素、依托泊甙、环磷酰胺、氮唏咪胺等，化疗2个-6个周期。

### 1.3 统计学处理方法

采用SPSS 16.0软件进行统计学分析。生存期计算自手术日起至死亡时间或者末次随访日（2010年10月）。用*Kaplan-Meier*曲线及*COX*回归分析性别、肿瘤大小、肿瘤分型及化疗对术后生存期的影响，*P* < 0.05为有统计学差异。

## 2 结果

### 2.1 临床特点

本组患者主要症状为咳嗽咳痰（7/20）、痰中带血（4/20）、胸痛（3/20）、胸闷（2/20）、发热等（2/20），有2例患者无任何症状和体征，为体检时发现。17例行痰细胞学检查，全部为阴性。12例行纤维支气管镜检查，1例管腔受压狭窄，但咬检结果为阴性。20例全部行胸部CT检查，中心型6例，周围型14例，右上肺3例，右中叶4例，右下肺6例，左上肺2例，左下肺5例，肿瘤大小2.0 cm-15.0 cm，直径5 cm以下的8例，直径在5 cm-10 cm之间的8例，10 cm以上的4例。14例边缘较规整，2例边缘可见毛刺征，1例见浅分叶，1例多发肺内转移，1例胸腔积液，1例侵犯肋骨。12例行碘普罗胺造影剂强化CT检查，均有中度强化，2例中心坏死。4例纵隔淋巴结肿大。

全部病例均经术后病理诊断为肺原发性MFH。其中，席纹状-多形性型16例，粘液型3例，炎性型1例。15例手术标本行免疫组化检查，结果为CD68(+)，Vimentin(+)。4例纵隔淋巴结肿大病例中2例有纵隔淋巴结转移。

### 2.2 术后生存率

全组无手术死亡。采用门诊定期复查、信访和电话相结合的方式，自手术日起随访至患者死亡或者截止日期2010年10月。中位随访期3年。全组生存期3个月-85个月，中位生存期12.5个月，1年、2年及3年生存率分别为55.0%、25.0%和10.0%。1例患者生存期达到85个月。患者死亡原因分别为：局部复发6例，胸膜转移2例，脑转移3例，全身广泛转移7例及其他原因2例。生存曲线见图 1所示，从该生存曲线可以看出，生存率在10个月-25个月间下降趋势最明显，说明肺原发性MFH患者术后在10个月-25个月间复发转移乃至死亡的风险最大。

**1:**
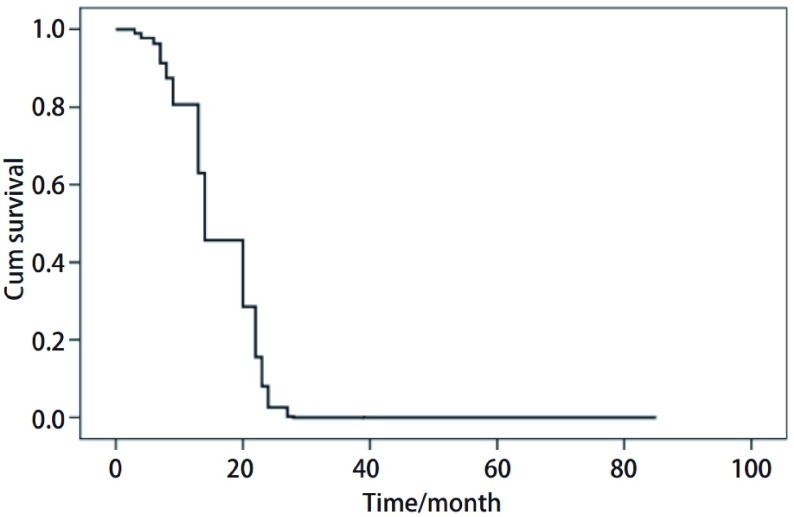
全组肺原发性MFH生存曲线 Survival curve of 22 MFH patients

### 2.3 影响肺原发MFH预后的因素分析

对全组肺原发MFH患者进行单因素分析，结果显示，影响其中位生存期的因素有肿瘤大小（χ^2^=12.255, *P*=0.002）及肿瘤分型（χ^2^=22.819, *P*=0.021），肿瘤 < 5 cm组2年生存率为50%，而肿瘤≥5 cm组2年生存率为8.3%；周围型和中心型2年生存率分别为28.6%和6.7%。患者性别、化疗对中位生存期无明显影响（表 1）。对影响肺原发MFH患者预后的单因素进行*COX*多因素综合分析（表 2），结果显示肿瘤大小及位置为肺原发MFH患者生存期的独立影响因素。

**1:** 影响肺原发MFH患者预后的单因素分析 Monofactorial analysis of prognosis of MFH patients

Factor	Case	Postoperative survival rate		Median survival (month)	*X*^2^	*P*
		1 year	2 year			
Sex					0.144	0.481
Male	15	53.3%	26.7%	12		
Female	5	60.0%	20.0%	20		
Adjuvant chemotherapy					0.109	0.383
Chemotherapy	9	55.6%	22.2%	12		
Non-chemotherapy	11	54.5%	27.3%	13		
Mass size					12.255	0.002
< 5 cm	8	75%	50%	27		
≥5 cm	12	41.7%	8.3%	8		
Site of mass					22.819	0.021
Central	6	33.3%	6.7%	6		
Peripheral	14	64.3%	28.6%	23		

**2:** 影响肺原发MFH患者生存期的多因素分析 Multivariate *COX* regression analysis of OS of MFH patients

Correlative factor	*P*	SE	*P*	Exp(B)	95%CI
Mass size	2.024	0.860	0.019	7.569	1.402-40.875
Site of mass	-2.875	1.120	0.010	0.056	0.060-0.506

## 3 讨论

恶性纤维组织细胞瘤是一种高度恶性的起源于间叶组织的肿瘤，首先由O’Brien等^[6]^在1964年作为一种独立类型的恶性肿瘤来进行描述。MFH好发于四肢、躯干及腹膜后，是成人最常发生的肉瘤之一，约占所有软组织肉瘤的10%。虽然肺脏是MFH最容易转移的器官之一，但是肺原发性MFH非常罕见，文献报道期发病率约为肺原发恶性肿瘤的0.02%-0.30%^[3]^，我院胸外科从1986年-2008年间共收治肺部恶性肿瘤13, 000余例，肺原发MFH占肺部恶性肿瘤的0.21%。肺原发MFH以中老年男性多见，儿童病例尚未见报道。本组患者中位年龄为61.6岁，男性患者占75.0%。肺原发MFH常见的临床症状有咳嗽咳痰、咯血（痰中带血）、胸痛、胸闷及发热等，一些少见的症状，如肺栓塞、低血糖、肥大性肺性骨关节病及粒细胞缺乏症等也有所报道^[7]^，但这些均无特异性，加之影像学检查也无特异性表现，因此，一般术前较难诊断，容易误诊为原发性肺癌，本组病例术前均未能确诊，有18例术前考虑为原发性肺癌。MFH起源于间叶组织，在气管及肺泡外生长，因此，痰脱落细胞学检查及气管镜刷片细胞学检查结果一般为阴性。

本组患者肿瘤大小2.0 cm-15 cm，直径5 cm的占60%，周围型占70%，大多为形态规则界限较清楚的实性肿物，这可能与肿瘤生长速度较快，成膨胀性生长形成假包膜有关。肺原发MFH较少发生纵隔及肺门淋巴结转移，本组仅4例出现纵隔淋巴结肿大，术后病理证实仅2例阳性，与文献报道较为一致。MFH容易侵犯邻近组织及脏器，本组患者有1例发生胸膜转移，1例肺内多发转移，1例侵犯肋骨。12例行强化CT检查发现均有中度强化。总结本组病例，肺原发性MFH的一些影像学特点可能有助于术前临床诊断：瘤体较大的实性肿物；以中下叶多见、多为外周型；边缘较光滑形态较规整；大多呈中度强化；较少发生纵隔淋巴结转移。

肺原发MFH显微镜下以席纹状的多形性纤维细胞及组织细胞为特征，其病理表现和其它部位的MFH无明显差异，其组织起源被认为是原始的间质细胞向不同方向分化形成。MFH缺乏特异性抗原，但是MFH瘤细胞一般对CD68、Vimentin呈阳性反应，而对CK、Actin、S100、Desmin呈阴性反应^[1]^，可以用免疫组化鉴别MFH和一些其它的软组织肉瘤。MFH的诊断需要取材充分，一般的穿刺取材较难和其它软组织肉瘤鉴别^[3]^。

肺原发MFH较少发生淋巴结转移，血行转移较常见，患者死因多为远处转移或者局部复发。最容易发生远处转移的器官为脑和骨。肺原发MFH的预后较差，文献报道相差较大，徐志龙等^[8]^报道肺原发MFH的1年生存率为60%，3年生存率为42%，5年生存率为33%；而Rzyman等^[9]^报道大部分患者于术后1年内死亡。本组患者1年、3年及5年生存率分别为55.0%、10.0%和5.0%，大部分患者于2年内死亡，患者死亡原因分别为远处转移和局部复发。有学者^[10]^认为术后辅助放疗的作用有限，但是可用于病变侵及胸壁及非根治性手术的术后治疗。另有学者^[11]^认为术后辅助化疗有助于延长患者生存期。本组患者进行单因素分析后发现术后辅助化疗并没有明显改善预后。鉴于病例数较少，更确切的结论需要多中心大样本进一步研究。MFH的主要治疗手段目前仍然是手术，改善预后关键在于早期发现及及时治疗^[12]^。本组病例发现， < 5 cm组的预后明显较≥5 cm组的好，多因素*COX*分析肿瘤大小为影响预后的独立因素，这可能是因为瘤体较小一般说明病程仍处于早期，发生侵犯附近脏器和远处转移的几率较小，根治的可能性较大。肿瘤的位置是另一个影响预后的独立因素，周围型较中央型患者中位生存期明显延长。这可能是因为中央型患者更容易早期侵犯气管、大血管等重要脏器。

综上所述，肺原发MFH是一种高度恶性的肉瘤，预后差。无特异性临床表现，早期诊断较困难。早期行根治性手术是取得较好疗效的关键，辅助性放化疗疗效尚不确切。
